# Synthesis and structure of (*RS*)-6-hy­droxy-6-(2-oxoprop­yl)-1,10-phenanthrolin-5(6*H*)-one

**DOI:** 10.1107/S2056989026004408

**Published:** 2026-05-07

**Authors:** Tarek Benlatreche, Abderrahmane Mezrag, Boutheina Boualia, Stéphane Golhen

**Affiliations:** a Environmental and Structural Molecular Chemistry Research Unit URCHEMS Faculty of Exact Sciences University of Constantine 1-Mentouri Brothers 25000 Algeria; b National Higher School for Hydraulics, Abdellah Arbaoui, Blida, Algeria; c Research Unit Development of Natural Resources, Bioactive Molecules and Physiochemical and Biological Analysis, Department of Chemistry, Constantine 1 University, Constantine 25000, Algeria; d Faculty of Science Department of Organic Chemistry Saad Dahleb University, Blida 1 Algeria; e CNRS Rennes Institute of Chemical Sciences -UMR 6226 University of Rennes France; University of Aberdeen, United Kingdom

**Keywords:** crystal structure, phenanthroline-5,6-dione derivative, O—H⋯N and C—H⋯O hydrogen bonds, Hirshfeld surface analysis

## Abstract

The title compound, C_15_H_12_N_2_O_3_, was synthesized by reacting phendione with acetone in ethanol under microwave irradiation. An intra­molecular C=O⋯π inter­action supports the mol­ecular conformation. In the crystal, inversion dimers linked by pairwise, bifurcated O—H⋯(N,N) hydrogen bonds are seen and the dimers are further linked by weak C—H⋯N and C—H⋯O hydrogen bonds and aromatic π–π stacking inter­actions. Hirshfeld surface analysis shows the following contact percentages: H⋯H 36.8%; H⋯O/O⋯H 26.1%; H⋯C/C⋯H 14.9%; H⋯N/N⋯H 14.5%; C⋯C 5.3%, with all other contact types making negligible contributions.

## Abstract 2

The title compound was synthesized by reacting phendione with acetone in ethanol under microwave irradiation. An intra­molecular C=O⋯π inter­action supports the mol­ecular conformation. In the crystal, inversion dimers linked by pairwise, bifurcated O—H⋯(N,*N*) hydrogen bonds are seen and the dimers are further linked by weak C—H⋯N and C—H⋯O hydrogen bonds and aromatic π–π stacking inter­actions.

## 1. Chemical context

1,10-Phenanthroline-5,6-dione (phendione, C_12_H_6_N_2_O_2_) is a quinonoid derivative of 1,10-phenanthroline, characterized by the presence of two carbonyl groups at positions 5 and 6 of the aromatic core, which confers both di­imine- and quinone-type reactive sites. This dual functionality provides significant versatility in coordination chemistry, allowing it to bind to metal ions primarily through the nitro­gen atoms of the di­imine moiety (Ermakova *et al.*, 2023 ▸), while in certain cases also engaging the oxygen atoms of the carbonyl groups in the coordination process (Jing *et al.*, 2011 ▸).
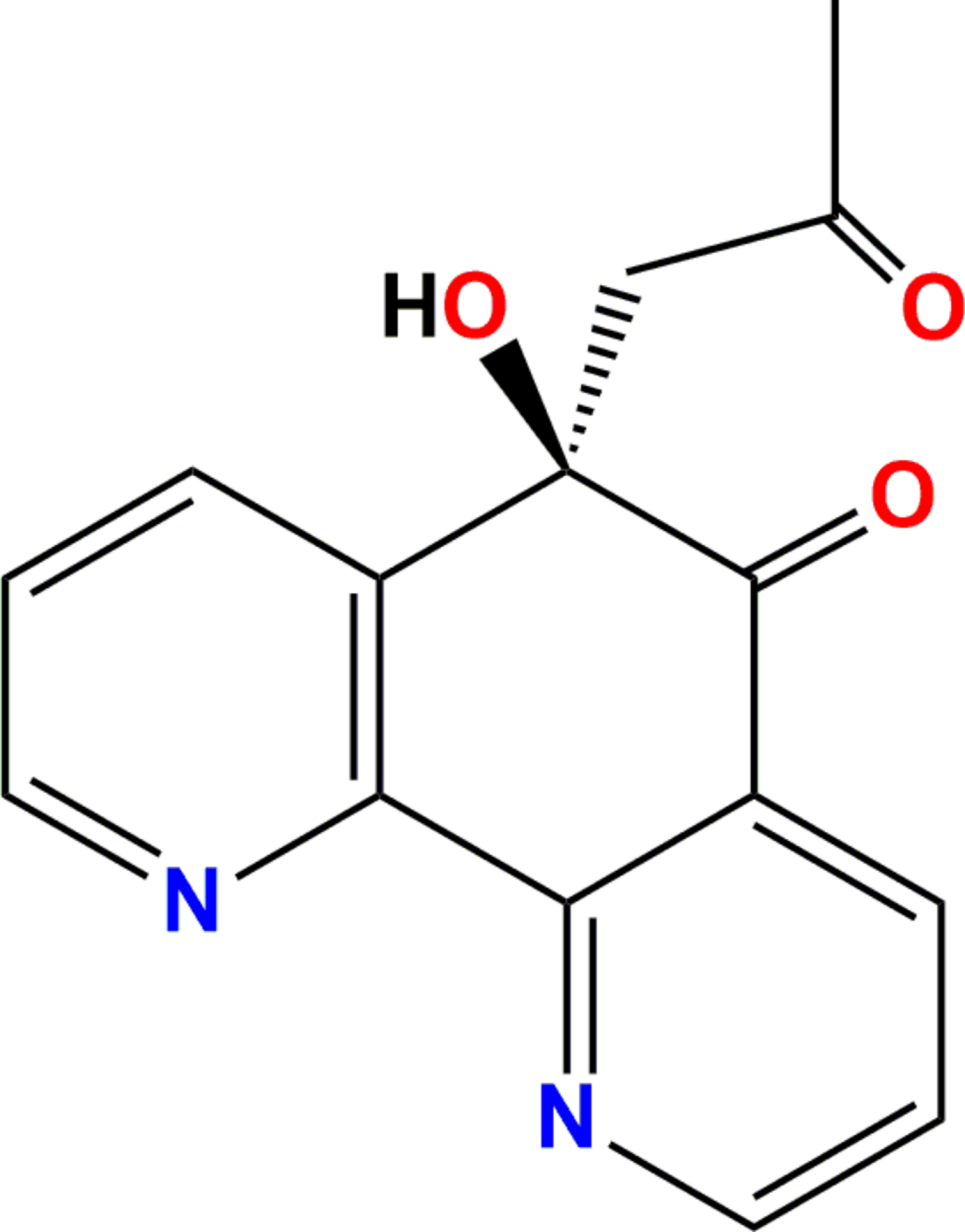


Phendione and its derivatives have broad applications in biology (Pivetta *et al.*, 2014 ▸; McCann *et al.*, 2012 ▸), chemistry, and medicinal chemistry. Their complexes, particularly those formed with Cu^II^ and Ag^I^, exhibit anti­microbial (Galdino *et al.*, 2022 ▸), anti­fungal (Granato *et al.*, 2017 ▸) and anti­tumor activities (Deegan *et al.*, 2006 ▸) due to their ability to inter­act with DNA and disrupt cellular redox processes (Pivetta *et al.*, 2014 ▸). They thus target both cancer cells and drug-resistant bacteria, making them promising candidates for the development of new therapeutic agents (Granato *et al.*, 2021 ▸).

As part of our studies in this area, we now describe the synthesis and structure of the title compound, C_15_H_12_N_2_O_3_ (**I**). We are particularly interested in this molecule because of its promising biological properties observed in our previous investigations, especially its anticholinesterase and antifungal activities against several tested strains. In addition, this compound exhibits a strong ability to coordinate with metal ions due to the presence of suitable donor atoms in its structure. In our work, special attention is given to its interaction with tin, as organotin derivatives are known to exhibit enhanced biological activities. Therefore, the synthesis and characterization of such complexes are of particular interest, not only to evaluate their potential biological properties, but also to gain deeper insight into the structure–activity relationships. This approach is fully consistent with our research objectives, which focus on the development of new bioactive compounds through coordination chemistry.

## 2. Structural commentary

**Figure 1:**
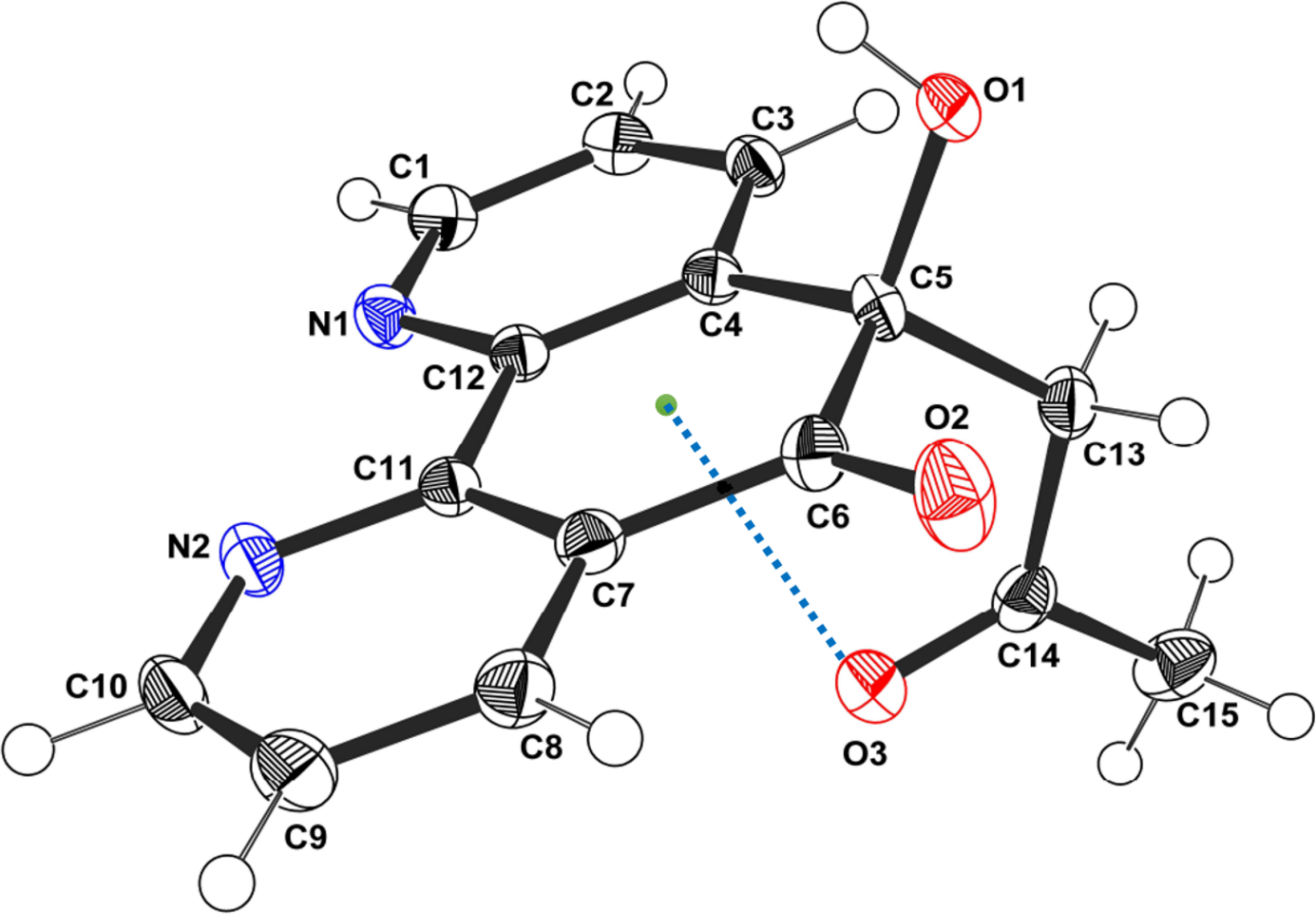
The asymmetric unit of (**I**) with displacement ellipsoids drawn at the 50% probability level.

Compound (**I**) crystallizes in the monoclinic space group *P*2_1_/*c* with one mol­ecule in the asymmetric unit (Fig. 1 ▸). The acetone moiety is attached to the aromatic core *via* atom C13 and adopts an approximately planar conformation, as indicated by the O3—C14—C13—C5 torsion angle of −11.3° (2), which may be associated with an intra­molecular C14=O3⋯π inter­action with O⋯*Cg* = 2.7130 (16) Å and C=O⋯π = 98.71 (11)°. The dihedral angle between the acetone group and the phenanthroline ring system is 84.99 (5)°, reflecting an almost perpendicular orientation. Bond lengths in the mol­ecule vary from 1.211 (2) Å (O3—C14) to 1.545 (3) Å (C5—C6), while bond angles range from 107.86 (14)° (O1—C5—C6) to 124.13 (18)° (N2—C10—C9). The mol­ecule features a quasi-planar aromatic core, whereas the carbonyl groups and peripheral substituents adopt moderate distortions to reduce steric inter­actions, as illustrated by the torsion angles C3—C4—C5—O1 = 58.65 (19)°, C3—C4—C5—C13 = −57.3 (2)°, C12—C4—C5—O1 = −118.38 (16)° and C12—C4—C5—C13 = 125.62 (17)°. Atom C5 is a stereogenic (chiral) centre: in the arbitrarily-chosen asymmetric unit, it has *R* configuration, but crystal symmetry generates a racemic mixture.

## 3. Supra­molecular features

**Figure 2:**
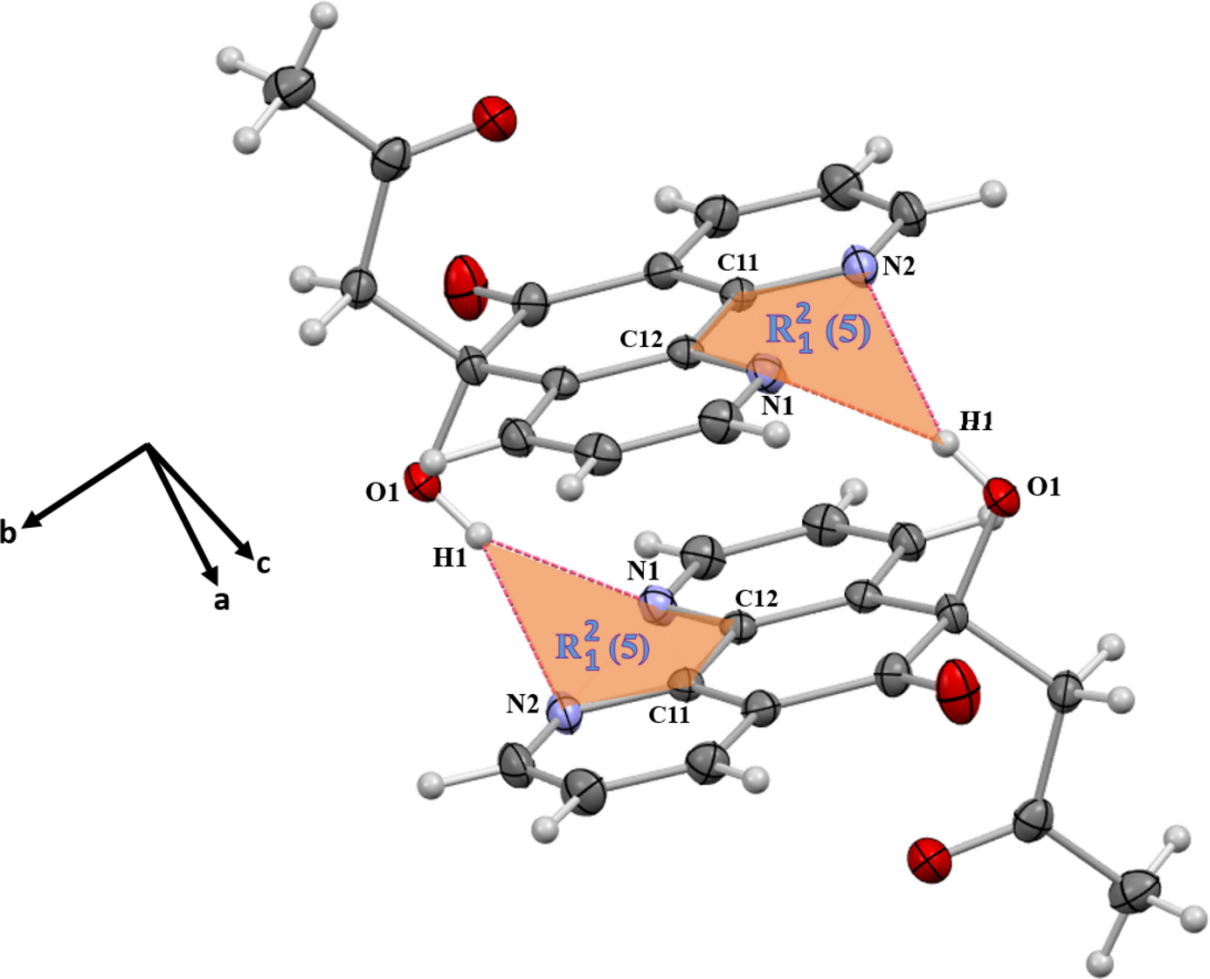
Detail of the packing of (**I**) illustrating the bifurcated O1—H1⋯(N1,N2) hydrogen bonds, which form an inversion dimer.

**Figure 3:**
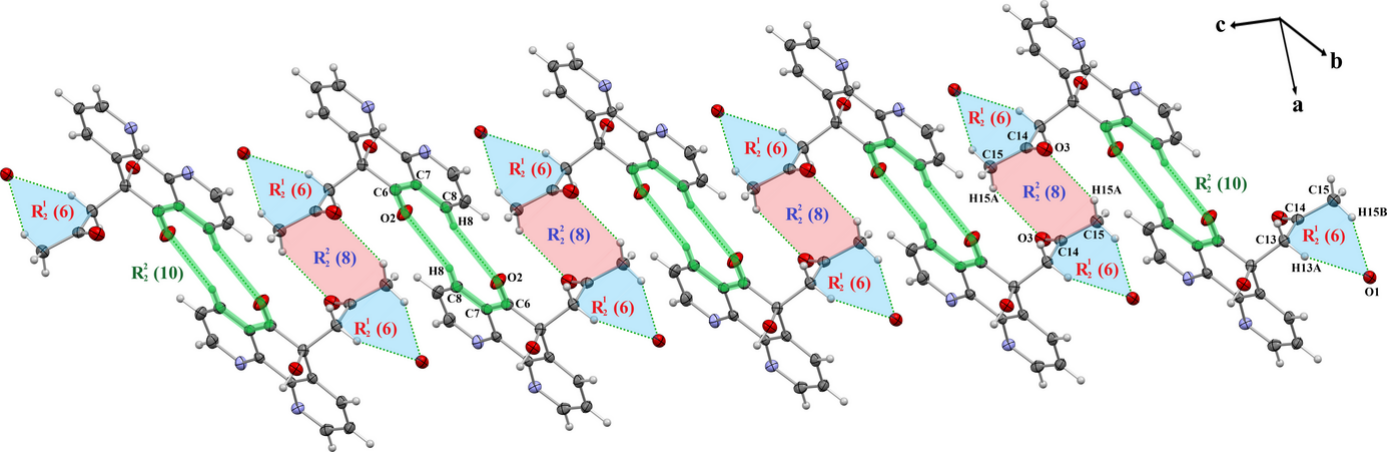
Detail of the packing of (**I**) showing the C15—H15B⋯O1, C13—H13*A*⋯O1, C15—H15*A*⋯O3 and C8—H8⋯O2 hydrogen bonds, forming different ring motifs.

**Figure 4:**
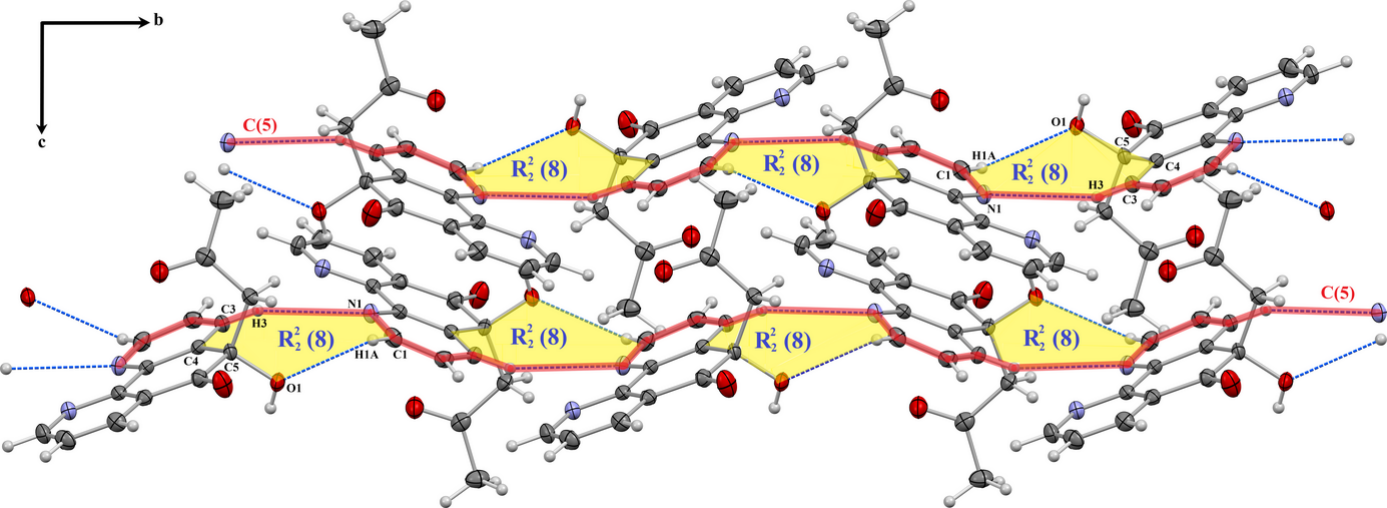
Crystal packing of (**I**) illustrating the C1—H1A⋯O1 and C3—H3⋯N1 hydrogen bonds forming a ring motif; and *C*(5) chains extending parallel to the *b-*axis direction, respectively.

In the extended structure of (**I**), an asymmetric, bifurcated O1—H1⋯(N1,N2) inter­action, with H⋯*A* distances of 2.22 and 2.56 Å, respectively (sum of angles at H1 = 359°), gives rise to an 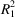
(5) ring motif, linking the mol­ecules into inversion dimers (Table 1 ▸, Fig. 2 ▸). The hydrogen bonds C13—H13*A*⋯O1 and C15—H15*B*⋯O1, with H⋯*A* distances of 2.52 and 2.67 Å, respectively, generate 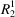 (6) loops connecting adjacent mol­ecules, which propagate along the *c*-axis direction (Fig. 3 ▸). Two 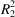
(8) motifs are also observed: the first is formed by C1—H1*A*⋯O1 and C3—H3⋯N1 (H⋯*A* = 2.63 and 2.83 Å; Fig. 4 ▸), while the second arises from C15—H15*A*⋯O3 (H⋯*A* = 2.69 Å; Fig. 3 ▸). These motifs repeat along the *b* and *c* axes, respectively, contributing to the long-range organization of the crystal. The C8—H8⋯O2 inter­action (H⋯*A* = 2.62 Å) forms an 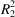
(10) ring motif linking two neighboring mol­ecules, propagating along the *c*-axis direction (Fig. 3 ▸). Additionally, the C3—H3⋯N1 bond generates *C*(5) chains extending parallel to the *b-*axis direction (Fig. 4 ▸), further reinforcing the continuity of hydrogen-bonding inter­actions within the structure.

**Figure 5:**
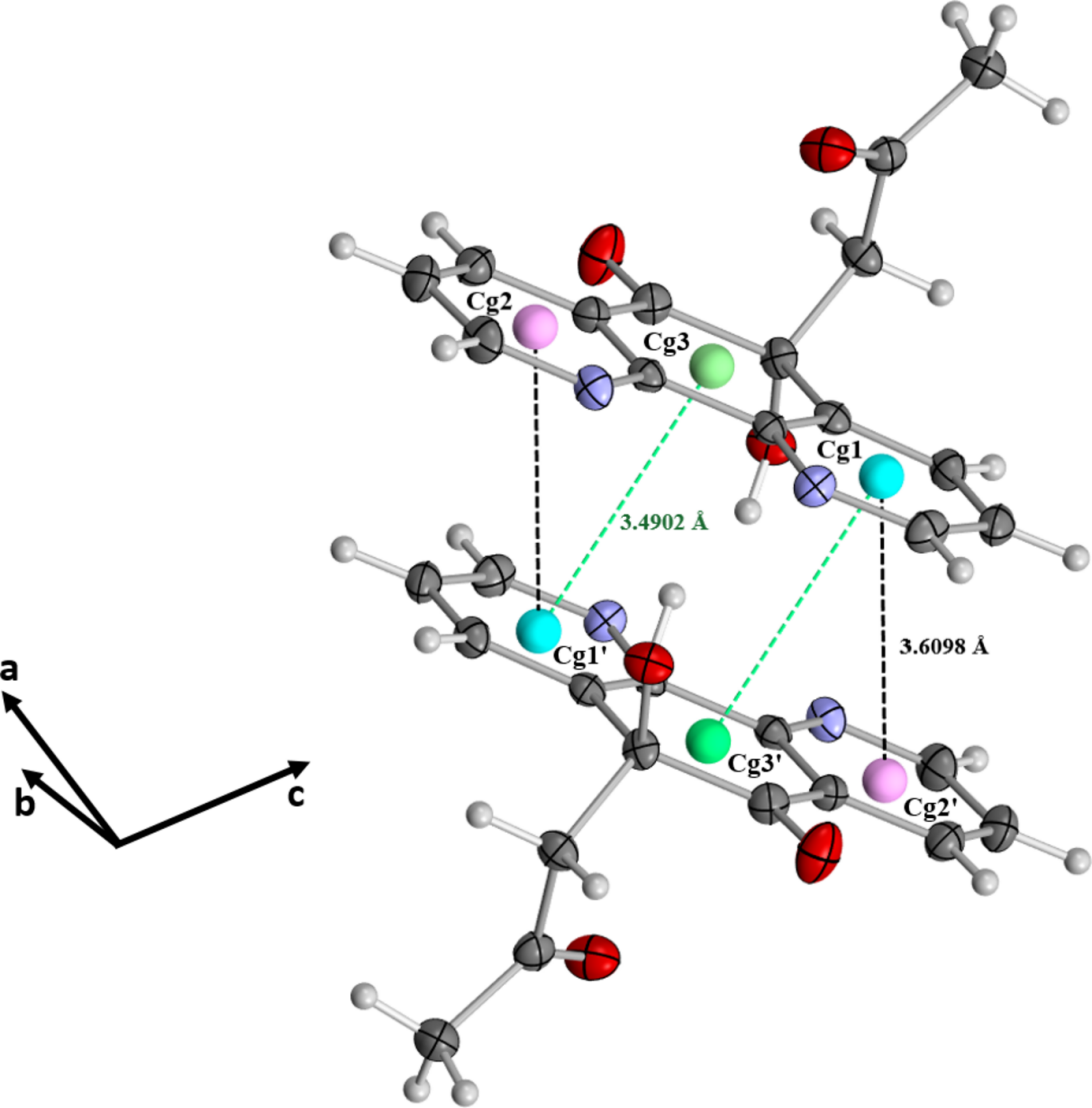
Depiction of π–π stacking inter­actions between the aromatic rings of superposed mol­ecules, with centroid–centroid distances (*Cg*1⋯*Cg*2′/*Cg*1⋯*Cg*3′) highlighted.

The three-dimensional architecture is consolidated by aromatic π–π stacking inter­actions between superposed mol­ecules. The centroid–centroid distances are 3.6098 (12) and 3.4902 (11) Å, observed between the ring centroids *Cg*1 and *Cg*2′ [symmetry code: (′) 1 − *x*, 1 − *y*, −*z*], where *Cg*1 and *Cg*2 correspond to the N1/C1–C4/C12 and N2/C7–C10/C11 rings, respectively, as well as *Cg*1 and *Cg*3′, where *Cg*3 represents the centroid of the C11/C4–C7/C12 ring (Fig. 5 ▸).

## 4. Database survey

A search of the Cambridge Structural Database (CSD, version 2025.3.1, update of February 2026; Groom *et al.*, 2016 ▸) for compounds similar to (**I**) was undertaken.

Database analysis revealed that the structure of (**I**) had not been reported previously, although similar structures were identified in monoatomic ruthenium(II), copper(II), and tin(IV) complexes. These structures, with CSD refcodes ATOPUU (Fujihara *et al.*, 2004 ▸), RUZQEJ (Karnahl *et al.*, 2010 ▸) and TILQOY (Benlatreche, 2023 ▸), crystallize in space groups *C*2/*c*, *P*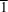 and *Pna*2_1_, respectively. Furthermore, another ligand, formed in a distinct complex (JIVPOX; Golubeva *et al.*, 2023 ▸), exhibits a structure similar to our mol­ecule, with the difference that the acetone group is replaced by an eth­oxy group. This complex crystallizes in space group *P*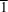.

## 5. Hirshfeld surface analysis

In order to further qu­antify the inter­molecular inter­actions contributing to the organization of the crystal packing, a Hirshfeld surface (HS) analysis, accompanied by an analysis of the associated two-dimensional fingerprint plots (FP), was carried out using *CrystalExplorer 21.5* (Spackman *et al.*, 2021 ▸).

**Figure 6:**
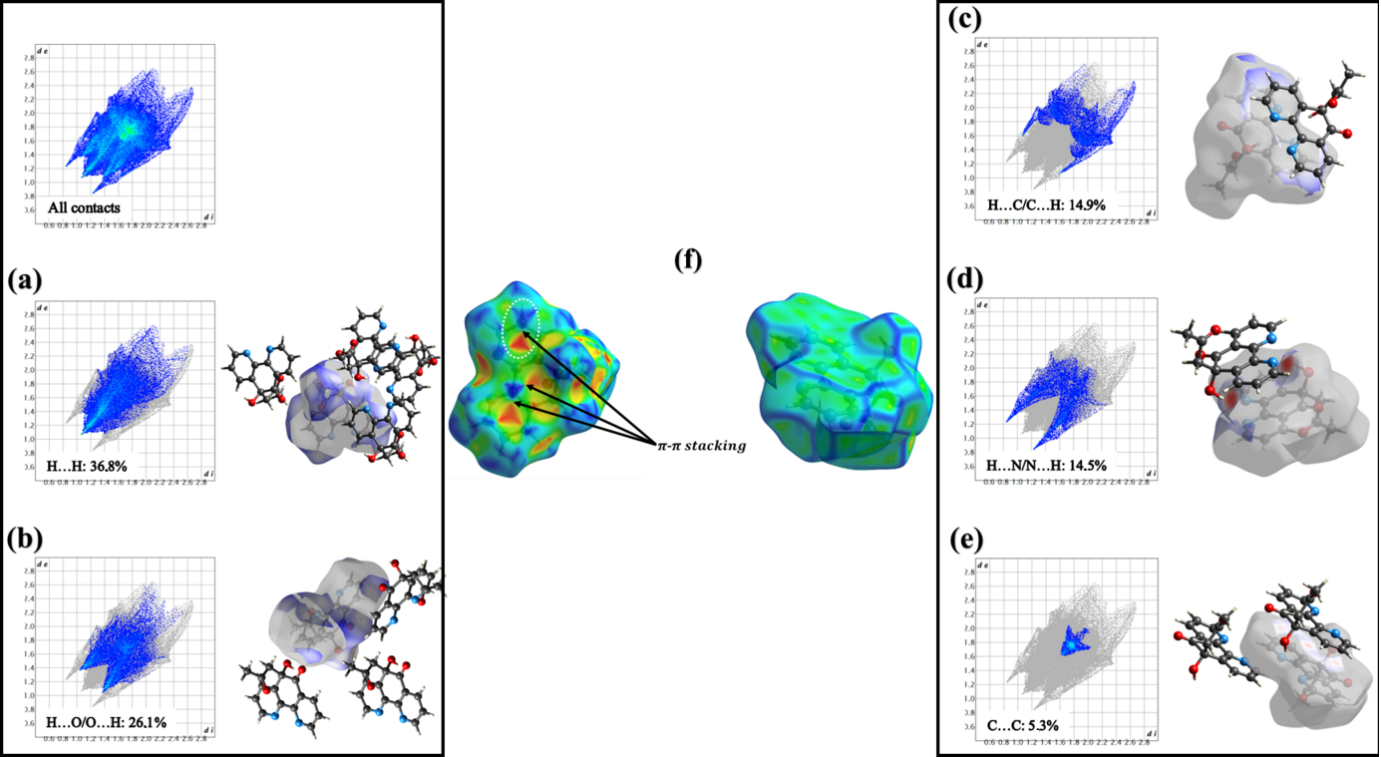
Hirshfeld surface analysis and two-dimensional fingerprint plots of the title compound illustrating: (*a*) H⋯H contacts, (*b*) O⋯H/H⋯O contacts, (*c*) H⋯C/C⋯H contacts, (*d*) N⋯H/H⋯N contacts, (*e*) C⋯C contacts; and Hirshfeld surface representations of the (*f*) shape-index and curvedness, highlighting π–π stacking inter­actions.

The Hirshfeld *d*_norm_ surfaces were mapped over the range −0.41 to 1.36 Å using a fixed colour scale from 0.76 (red) to 2.4 (blue). The Hirshfeld surface was further investigated through the associated two-dimensional fingerprint plots, which provide a qu­anti­tative representation of the inter­molecular contacts within the crystal structure. As shown in Fig. 6 ▸*a*, H⋯H contacts constitute the major contribution to the Hirshfeld surface, accounting for 36.8% of the total surface area. These contacts mainly arise from C—H⋯H inter­actions and emphasize the predominance of van der Waals forces in the crystal packing. The O⋯H/H⋯O contacts, shown in Fig. 6 ▸*b*, correspond to C—H⋯O hydrogen-bond inter­actions and represent the second most important contribution, accounting for 26.1% of the total inter­actions.

Fig. 6 ▸*c* displays the H⋯C/C⋯H contacts, which are associated with C—H⋯π inter­actions and contribute 14.9% to the Hirshfeld surface. In addition, the N⋯H/H⋯N contacts (Fig. 6 ▸*d*), attributable to O—H⋯N hydrogen bonds, represent 14.5% of the total surface area, highlighting the role of these inter­actions in the supra­molecular assembly. The C⋯C contacts illustrated in Fig. 6 ▸*e* account for 5.3% of the total inter­actions and are indicative of π–π stacking inter­actions between aromatic rings (Fig. 6 ▸*f*). Other inter­molecular contacts appear only as minor contributions in the fingerprint plots, including N⋯C/C⋯N (1.7%), O⋯C/C⋯O (0.5%) and O⋯O (0.2%) contacts.

## 6. Synthesis and crystallization

In a 10 ml glass vial, 0.25 mmol of 1,10-phenanthroline-5,6-dione was combined with an equimolar mixture of ethanol and acetone, filling approximately two-thirds of the vial. The vial was then placed in a microwave and irradiated at 393 K for 2 minutes. Following the addition of acetone, colourless crystals of (**I**) were obtained after 15 days at room temperature. Yield: 90%

## 7. Refinement

Crystal data, data collection and structure refinement details are summarized in Table 2 ▸. Hydrogen atoms were positioned geometrically and allowed to ride on their parent atoms with C—H = 0.95–0.99 Å and O—H = 0.84 Å. The constraint *U*_iso_(H) = 1.2*U*_eq_(C) or 1.5*U*_eq_(methyl C or O) was applied in all cases.

## Supplementary Material

Crystal structure: contains datablock(s) I. DOI: 10.1107/S2056989026004408/hb8213sup1.cif

Structure factors: contains datablock(s) I. DOI: 10.1107/S2056989026004408/hb8213Isup2.hkl

Supporting information file. DOI: 10.1107/S2056989026004408/hb8213Isup3.cml

CCDC reference: 2549869

Additional supporting information:  crystallographic information; 3D view; checkCIF report

## Figures and Tables

**Table 1:** Hydrogen-bond geometry (Å, °)

*D*—H⋯*A*	*D*—H	H⋯*A*	*D*⋯*A*	*D*—H⋯*A*
O1—H1⋯N1^i^	0.84	2.22	2.977 (2)	149
O1—H1⋯N2^i^	0.84	2.56	3.262 (2)	142
C13—H13*A*⋯O1^ii^	0.99	2.52	3.457 (2)	158
C15—H15*B*⋯O1^ii^	0.98	2.67	3.561 (3)	152
C1—H1*A*⋯O1^iii^	0.95	2.63	3.302 (2)	128
C3—H3⋯N1^iii^	0.95	2.83	3.727 (2)	158
C8—H8⋯O2^iv^	0.95	2.62	3.436 (3)	145
C10—H10⋯O3^v^	0.95	2.59	3.235 (2)	125
C15—H15*A*⋯O3^vi^	0.98	2.69	3.254 (3)	117

Symmetry codes: (i) 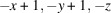; (ii) 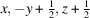; (iii) 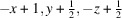; (iv) 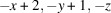; (v) 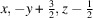; (vi) 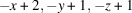.

**Table 2:** Experimental details

Crystal data	
Chemical formula	C_15_H_12_N_2_O_3_
*M*_r_	268.27
Crystal system, space group	Monoclinic, *P*2_1_/*c*
Temperature (K)	150
*a*, *b*, *c* (Å)	11.2440 (19), 12.604 (2), 8.9926 (14)
β (°)	106.870 (6)
*V* (Å^3^)	1219.6 (3)
*Z*	4
Radiation type	Mo *K*α
μ (mm^−1^)	0.10
Crystal size (mm)	0.25 × 0.2 × 0.18

Data collection	
Diffractometer	D8 VENTURE Bruker AXS
Absorption correction	Multi-scan (*SADABS*; Krause *et al.*, 2015 ▸)
No. of measured, independent and observed [*I* > 2σ(*I*)] reflections	9853, 2781, 2205
*R*_int_	0.046
(sin θ/λ)_max_ (Å^−1^)	0.650

Refinement	
*R*[*F*^2^ > 2σ(*F*^2^)], *wR*(*F*^2^), *S*	0.052, 0.132, 1.03
No. of reflections	2781
No. of parameters	183
H-atom treatment	H-atom parameters constrained
Δρ_max_, Δρ_min_ (e Å^−3^)	0.30, −0.28

Computer programs: *APEX3* and *SAINT* (Bruker, 2015 ▸), *SHELXT2018/2* (Sheldrick, 2015*a* ▸), *SHELXL2018/3* (Sheldrick, 2015*b* ▸) and *OLEX2* (Dolomanov *et al.*, 2009 ▸).

## supplementary crystallographic information

### (RS)-6-Hydroxy-6-(2-oxopropyl)-1,10-phenanthrolin-5(6H)-one Crystal data

**Table:**

C_15_H_12_N_2_O_3_	*F*(000) = 560
*M**_r_* = 268.27	*D*_x_ = 1.461 Mg m^−^^3^
Monoclinic, *P*2_1_/*c*	Mo *K*α radiation, λ = 0.71073 Å
*a* = 11.2440 (19) Å	Cell parameters from 6286 reflections
*b* = 12.604 (2) Å	θ = 2.5–27.5°
*c* = 8.9926 (14) Å	µ = 0.10 mm^−^^1^
β = 106.870 (6)°	*T* = 150 K
*V* = 1219.6 (3) Å^3^	Prism, colourless
*Z* = 4	0.25 × 0.2 × 0.18 mm

### (RS)-6-Hydroxy-6-(2-oxopropyl)-1,10-phenanthrolin-5(6H)-one Data collection

**Table:**

D8 VENTURE Bruker AXS diffractometer	2205 reflections with *I* > 2σ(*I*)
Detector resolution: 10.4167 pixels mm^-1^	*R*_int_ = 0.046
rotation images scans	θ_max_ = 27.5°, θ_min_ = 2.5°
Absorption correction: multi-scan (SADABS; Krause *et al.*, 2015)	*h* = −14→14
	*k* = −13→16
9853 measured reflections	*l* = −11→11
2781 independent reflections	

### (RS)-6-Hydroxy-6-(2-oxopropyl)-1,10-phenanthrolin-5(6H)-one Refinement

**Table:**

Refinement on *F*^2^	Primary atom site location: dual
Least-squares matrix: full	Hydrogen site location: inferred from neighbouring sites
*R*[*F*^2^ > 2σ(*F*^2^)] = 0.052	H-atom parameters constrained
*wR*(*F*^2^) = 0.132	*w* = 1/[σ^2^(*F*_o_^2^) + (0.0414*P*)^2^ + 1.046*P*] where *P* = (*F*_o_^2^ + 2*F*_c_^2^)/3
*S* = 1.03	(Δ/σ)_max_ < 0.001
2781 reflections	Δρ_max_ = 0.30 e Å^−^^3^
183 parameters	Δρ_min_ = −0.28 e Å^−^^3^
0 restraints	

### (RS)-6-Hydroxy-6-(2-oxopropyl)-1,10-phenanthrolin-5(6H)-one Special details

**Table:**

Geometry. All esds (except the esd in the dihedral angle between two l.s. planes) are estimated using the full covariance matrix. The cell esds are taken into account individually in the estimation of esds in distances, angles and torsion angles; correlations between esds in cell parameters are only used when they are defined by crystal symmetry. An approximate (isotropic) treatment of cell esds is used for estimating esds involving l.s. planes.

### (RS)-6-Hydroxy-6-(2-oxopropyl)-1,10-phenanthrolin-5(6H)-one Fractional atomic coordinates and isotropic or equivalent isotropic displacement parameters (Å^2^)

**Table:**

	*x*	*y*	*z*	*U*_iso_*/*U*_eq_	
O1	0.66938 (12)	0.29055 (10)	0.12038 (15)	0.0220 (3)	
H1	0.606402	0.305120	0.046242	0.033*	
O3	0.84247 (13)	0.52378 (11)	0.43924 (16)	0.0299 (3)	
O2	0.88727 (13)	0.39743 (12)	0.10860 (18)	0.0333 (4)	
N1	0.47777 (13)	0.60754 (12)	0.17416 (17)	0.0194 (3)	
N2	0.63092 (14)	0.70543 (12)	0.03798 (17)	0.0208 (3)	
C12	0.57858 (15)	0.55159 (14)	0.16619 (18)	0.0152 (3)	
C11	0.66176 (15)	0.60569 (14)	0.08928 (18)	0.0165 (4)	
C7	0.76747 (15)	0.55389 (15)	0.07228 (19)	0.0183 (4)	
C4	0.60379 (15)	0.44875 (14)	0.22495 (19)	0.0161 (4)	
C3	0.52129 (16)	0.40246 (15)	0.29597 (19)	0.0194 (4)	
H3	0.533697	0.331769	0.333955	0.023*	
C5	0.71304 (16)	0.38418 (14)	0.20787 (19)	0.0174 (4)	
C6	0.79761 (16)	0.44393 (15)	0.1278 (2)	0.0200 (4)	
C10	0.70589 (18)	0.75471 (15)	−0.0311 (2)	0.0242 (4)	
H10	0.685095	0.824996	−0.067532	0.029*	
C14	0.84877 (15)	0.43219 (16)	0.4815 (2)	0.0215 (4)	
C8	0.84341 (16)	0.60787 (16)	−0.0011 (2)	0.0226 (4)	
H8	0.915370	0.574755	−0.014840	0.027*	
C13	0.79505 (16)	0.34433 (15)	0.3669 (2)	0.0206 (4)	
H13A	0.744812	0.296932	0.412625	0.025*	
H13B	0.864221	0.301854	0.350456	0.025*	
C1	0.40297 (17)	0.56254 (16)	0.2471 (2)	0.0226 (4)	
H1A	0.333471	0.601967	0.256368	0.027*	
C2	0.42114 (16)	0.46134 (16)	0.3101 (2)	0.0214 (4)	
H2	0.365825	0.432869	0.362005	0.026*	
C9	0.81238 (18)	0.70962 (17)	−0.0529 (2)	0.0267 (4)	
H9	0.862611	0.748262	−0.102620	0.032*	
C15	0.90883 (18)	0.39957 (18)	0.6461 (2)	0.0291 (5)	
H15A	0.988278	0.364693	0.653789	0.044*	
H15B	0.854171	0.350215	0.679350	0.044*	
H15C	0.923377	0.462428	0.713143	0.044*	

### (RS)-6-Hydroxy-6-(2-oxopropyl)-1,10-phenanthrolin-5(6H)-one Atomic displacement parameters (Å^2^)

**Table:**

	*U*^11^	*U*^22^	*U*^33^	*U*^12^	*U*^13^	*U*^23^
O1	0.0252 (7)	0.0134 (7)	0.0246 (7)	0.0021 (5)	0.0030 (5)	−0.0026 (5)
O3	0.0307 (7)	0.0206 (8)	0.0337 (7)	−0.0007 (6)	0.0021 (6)	0.0004 (6)
O2	0.0312 (7)	0.0280 (8)	0.0482 (9)	0.0095 (6)	0.0234 (7)	0.0086 (7)
N1	0.0195 (7)	0.0160 (8)	0.0235 (7)	0.0012 (6)	0.0072 (6)	−0.0008 (6)
N2	0.0263 (8)	0.0150 (8)	0.0210 (7)	0.0009 (6)	0.0065 (6)	0.0020 (6)
C12	0.0169 (8)	0.0147 (9)	0.0137 (7)	−0.0003 (6)	0.0041 (6)	−0.0020 (6)
C11	0.0185 (8)	0.0148 (9)	0.0148 (7)	−0.0011 (6)	0.0025 (6)	−0.0010 (6)
C7	0.0177 (8)	0.0192 (9)	0.0171 (8)	−0.0017 (7)	0.0035 (6)	−0.0003 (7)
C4	0.0182 (8)	0.0149 (9)	0.0146 (7)	−0.0001 (6)	0.0037 (6)	−0.0026 (6)
C3	0.0240 (9)	0.0153 (9)	0.0186 (8)	−0.0017 (7)	0.0058 (7)	0.0016 (7)
C5	0.0211 (8)	0.0116 (8)	0.0195 (8)	0.0013 (7)	0.0060 (6)	0.0026 (7)
C6	0.0200 (8)	0.0192 (10)	0.0213 (8)	0.0030 (7)	0.0068 (7)	0.0009 (7)
C10	0.0308 (10)	0.0162 (10)	0.0252 (9)	−0.0020 (8)	0.0073 (7)	0.0047 (7)
C14	0.0157 (8)	0.0257 (11)	0.0233 (9)	0.0027 (7)	0.0062 (7)	0.0025 (8)
C8	0.0195 (8)	0.0246 (10)	0.0242 (9)	−0.0007 (7)	0.0071 (7)	0.0019 (8)
C13	0.0224 (8)	0.0183 (10)	0.0195 (8)	0.0036 (7)	0.0038 (7)	0.0026 (7)
C1	0.0202 (8)	0.0229 (10)	0.0263 (9)	0.0013 (7)	0.0091 (7)	−0.0035 (8)
C2	0.0222 (9)	0.0239 (10)	0.0203 (8)	−0.0034 (7)	0.0098 (7)	−0.0013 (7)
C9	0.0265 (9)	0.0280 (11)	0.0270 (9)	−0.0052 (8)	0.0101 (7)	0.0061 (8)
C15	0.0252 (9)	0.0380 (13)	0.0223 (9)	−0.0042 (9)	0.0040 (7)	0.0019 (9)

### (RS)-6-Hydroxy-6-(2-oxopropyl)-1,10-phenanthrolin-5(6H)-one Geometric parameters (Å, º)

**Table:**

O1—H1	0.8400	C5—C6	1.545 (2)
O1—C5	1.424 (2)	C5—C13	1.543 (2)
O3—C14	1.211 (2)	C10—H10	0.9500
O2—C6	1.221 (2)	C10—C9	1.390 (3)
N1—C12	1.354 (2)	C14—C13	1.514 (3)
N1—C1	1.334 (2)	C14—C15	1.496 (2)
N2—C11	1.349 (2)	C8—H8	0.9500
N2—C10	1.336 (2)	C8—C9	1.375 (3)
C12—C11	1.481 (2)	C13—H13A	0.9900
C12—C4	1.398 (2)	C13—H13B	0.9900
C11—C7	1.403 (2)	C1—H1A	0.9500
C7—C6	1.479 (3)	C1—C2	1.386 (3)
C7—C8	1.398 (2)	C2—H2	0.9500
C4—C3	1.397 (2)	C9—H9	0.9500
C4—C5	1.518 (2)	C15—H15A	0.9800
C3—H3	0.9500	C15—H15B	0.9800
C3—C2	1.385 (3)	C15—H15C	0.9800
			
C5—O1—H1	109.5	C9—C10—H10	117.9
C1—N1—C12	117.48 (16)	O3—C14—C13	120.55 (16)
C10—N2—C11	117.24 (16)	O3—C14—C15	122.76 (18)
N1—C12—C11	115.89 (15)	C15—C14—C13	116.69 (17)
N1—C12—C4	122.95 (15)	C7—C8—H8	120.5
C4—C12—C11	121.15 (15)	C9—C8—C7	118.99 (17)
N2—C11—C12	117.01 (15)	C9—C8—H8	120.5
N2—C11—C7	122.56 (16)	C5—C13—H13A	108.8
C7—C11—C12	120.43 (16)	C5—C13—H13B	108.8
C11—C7—C6	121.18 (15)	C14—C13—C5	113.94 (15)
C8—C7—C11	118.54 (17)	C14—C13—H13A	108.8
C8—C7—C6	120.28 (16)	C14—C13—H13B	108.8
C12—C4—C5	122.71 (15)	H13A—C13—H13B	107.7
C3—C4—C12	118.12 (16)	N1—C1—H1A	118.2
C3—C4—C5	119.11 (16)	N1—C1—C2	123.56 (17)
C4—C3—H3	120.5	C2—C1—H1A	118.2
C2—C3—C4	118.91 (17)	C3—C2—C1	118.88 (16)
C2—C3—H3	120.5	C3—C2—H2	120.6
O1—C5—C4	109.93 (14)	C1—C2—H2	120.6
O1—C5—C6	107.86 (14)	C10—C9—H9	120.7
O1—C5—C13	105.05 (14)	C8—C9—C10	118.54 (17)
C4—C5—C6	114.29 (14)	C8—C9—H9	120.7
C4—C5—C13	111.36 (14)	C14—C15—H15A	109.5
C13—C5—C6	107.89 (14)	C14—C15—H15B	109.5
O2—C6—C7	121.31 (16)	C14—C15—H15C	109.5
O2—C6—C5	118.50 (16)	H15A—C15—H15B	109.5
C7—C6—C5	120.17 (15)	H15A—C15—H15C	109.5
N2—C10—H10	117.9	H15B—C15—H15C	109.5
N2—C10—C9	124.13 (18)		
			
O1—C5—C6—O2	−57.0 (2)	C11—C7—C8—C9	0.4 (3)
O1—C5—C6—C7	121.28 (16)	C7—C8—C9—C10	−0.4 (3)
O1—C5—C13—C14	−178.29 (14)	C4—C12—C11—N2	−179.14 (15)
O3—C14—C13—C5	−11.3 (2)	C4—C12—C11—C7	0.7 (2)
N1—C12—C11—N2	1.4 (2)	C4—C3—C2—C1	2.8 (3)
N1—C12—C11—C7	−178.76 (14)	C4—C5—C6—O2	−179.53 (16)
N1—C12—C4—C3	−0.5 (2)	C4—C5—C6—C7	−1.3 (2)
N1—C12—C4—C5	176.56 (15)	C4—C5—C13—C14	−59.34 (19)
N1—C1—C2—C3	−0.7 (3)	C3—C4—C5—O1	58.65 (19)
N2—C11—C7—C6	−179.12 (15)	C3—C4—C5—C6	−179.92 (14)
N2—C11—C7—C8	−0.2 (3)	C3—C4—C5—C13	−57.3 (2)
N2—C10—C9—C8	0.1 (3)	C5—C4—C3—C2	−179.43 (15)
C12—N1—C1—C2	−2.0 (3)	C6—C7—C8—C9	179.34 (16)
C12—C11—C7—C6	1.0 (2)	C6—C5—C13—C14	66.84 (18)
C12—C11—C7—C8	179.99 (15)	C10—N2—C11—C12	179.80 (15)
C12—C4—C3—C2	−2.3 (2)	C10—N2—C11—C7	−0.1 (2)
C12—C4—C5—O1	−118.38 (16)	C8—C7—C6—O2	−1.4 (3)
C12—C4—C5—C6	3.1 (2)	C8—C7—C6—C5	−179.59 (16)
C12—C4—C5—C13	125.62 (17)	C13—C5—C6—O2	56.0 (2)
C11—N2—C10—C9	0.1 (3)	C13—C5—C6—C7	−125.72 (16)
C11—C12—C4—C3	−179.94 (15)	C1—N1—C12—C11	−177.91 (15)
C11—C12—C4—C5	−2.9 (2)	C1—N1—C12—C4	2.6 (2)
C11—C7—C6—O2	177.55 (17)	C15—C14—C13—C5	168.82 (15)
C11—C7—C6—C5	−0.7 (2)		

### (RS)-6-Hydroxy-6-(2-oxopropyl)-1,10-phenanthrolin-5(6H)-one Hydrogen-bond geometry (Å, º)

**Table:**

*D*—H···*A*	*D*—H	H···*A*	*D*···*A*	*D*—H···*A*
O1—H1···N1^i^	0.84	2.22	2.977 (2)	149
O1—H1···N2^i^	0.84	2.56	3.262 (2)	142
C13—H13*A*···O1^ii^	0.99	2.52	3.457 (2)	158
C15—H15*B*···O1^ii^	0.98	2.67	3.561 (3)	152
C1—H1*A*···O1^iii^	0.95	2.63	3.302 (2)	128
C3—H3···N1^iii^	0.95	2.83	3.727 (2)	158
C8—H8···O2^iv^	0.95	2.62	3.436 (3)	145
C10—H10···O3^v^	0.95	2.59	3.235 (2)	125
C15—H15*A*···O3^vi^	0.98	2.69	3.254 (3)	117

Symmetry codes: (i) −*x*+1, −*y*+1, −*z*; (ii) *x*, −*y*+1/2, *z*+1/2; (iii) −*x*+1, *y*+1/2, −*z*+1/2; (iv) −*x*+2, −*y*+1, −*z*; (v) *x*, −*y*+3/2, *z*−1/2; (vi) −*x*+2, −*y*+1, −*z*+1.
